# Ovarian Disease

**Published:** 1889-10-26

**Authors:** 


### OVARIAN DISEASE.

Mary A. Dixon Jones, M.D., of Brooklyn, writes of "A hitherto undescribed disease of the ovary."† The symptoms of cases detailed are very much those we are accustomed to include under the terms ovarian irritation or ovarian dysmenorrhœa. After a trial of many things at the hands of many physicians, the ovaries were removed from several persons, and plates of the microscopical appearances of this new disease are given. The growth from one patient was examined by Prof. Prudden, of New York, and by Waldeyer, Berlin, who both pronounced it cancerous. But "from studies and drawings," Dr. Mary Jones does not agree with this, and states "it is a new formation of bloodvessels and blood corpuscles." In short, the authoress regards it as "endothelioma changing to angeioma and hæmatoma." When doctors disagree who shall decide? But certainly Dr. Mary Jones reasons out her case well and elaborately, and seems very successful in curing her patients, which she would scarcely accomplish were they afflicted with cancer.

† *New York Medical Journal*, Sept. 28, 1889.

